# EGF-induced sodium influx regulates EGFR trafficking through HDAC6 and tubulin acetylation

**DOI:** 10.1186/s12860-015-0070-8

**Published:** 2015-09-18

**Authors:** Seung Joon Lee, Zhiqin Li, Alisa Litan, Soonmoon Yoo, Sigrid A. Langhans

**Affiliations:** Nemours Center for Childhood Cancer Research, Alfred I. duPont Hospital for Children, 1701 Rockland Road, Wilmington, DE 19803 USA

**Keywords:** Histone deacetylase, Na,K-ATPase, sodium, tubulin, acetylation, EGFR

## Abstract

**Background:**

Endocytosis of activated EGF receptor (EGFR) to specific endocytic compartments is required to terminate EGF signaling. Trafficking of EGFR relies on microtubule tracks that transport the cargo vesicle to their intermediate and final destinations and can be modulated through posttranslational modification of tubulin including acetylation. Na,K-ATPase maintains intracellular sodium homeostasis, functions as a signaling scaffold and interacts with EGFR. Na,K-ATPase also binds to and is regulated by acetylated tubulin but whether there is a functional link between EGFR, Na,K-ATPase and tubulin acetylation is not known.

**Results:**

EGF-induced sodium influx regulates EGFR trafficking through increased microtubule acetylation. Increased sodium influx induced either by sodium ionophores or Na,K-ATPase blockade mimicked the EGF-induced effects on EGFR trafficking through histone deacetylase (HDAC) 6 inactivation and accumulation of acetylated tubulin. In turn, blocking sodium influx reduced tubulin acetylation and EGF-induced EGFR turnover. Knockdown of HDAC6 reversed the effect of sodium influx indicating that HDAC6 is necessary to modulate sodium-dependent tubulin acetylation.

**Conclusions:**

These studies provide a novel regulatory mechanism to attenuate EGFR signaling in which EGF modulates EGFR trafficking through intracellular sodium-mediated HDAC6 inactivation and tubulin acetylation.

**Electronic supplementary material:**

The online version of this article (doi:10.1186/s12860-015-0070-8) contains supplementary material, which is available to authorized users.

## Background

EGF signaling triggers diverse molecular cascades to promote cytoskeletal reorganization, cell cycle progression and gene expression. Activation of EGF signaling is initiated by interaction of the ligand with a member of the EGFR family. Dimerization of EGFR induced by ligand binding leads to activation of the kinase activity with subsequent phosphorylation of downstream effectors including the receptor itself. The signal can be down-regulated through internalization of the activated receptor followed by selective lysosomal degradation. Upon clathrin-dependent endocytosis, endosomal vesicles containing the EGF-EGFR complex are delivered to early and late endosomes through interaction with motor proteins along the microtubule track. Vesicle trafficking along these tracks not only depends on the motor-cargo complex but can also be modulated through posttranslational modification of tubulin, including acetylation [1].

Like most acetylated proteins, tubulin acetylation is regulated by the balanced activity of histone acetyltransferases (HATs) and histone deacetylases (HDACs) that add and remove acetyl groups to lysine residues, respectively [2]. In addition to their well characterized role as transcriptional repressors, HDACs are known to have, depending on the isoform, many non-histone protein substrates, including transcription factors, hormone receptors, signaling mediators, chaperones, and cytoskeletal proteins [3, 4]. Class I HDACs, which are ubiquitously expressed and primarily localized to the nucleus, are crucial for transcriptional repression and epigenetic landscaping. Class II HDACs, which have a more tissue specific expression pattern, are subdivided into class IIa and IIb HDACs. Class IIa and IIb HDACs are able to shuttle between the cytosol and nucleus, but class IIb HDACs are mostly found in the cytosol with a preference for non-histone proteins. HDAC6 belongs to the class IIb HDACs and was the first enzyme known to deacetylate α-tubulin that undergoes reversible acetylation/deacetylation in the microtubule polymer, preferentially at lysine 40 [5]. While it remains controversial whether the acetylation of microtubules directly affects microtubule stability or microtubule binding to motor proteins [6], HDAC6 has been suggested to bind to EGFR and regulate EGFR endocytic trafficking and degradation through modulation of tubulin acetylation [1, 7]. However, the molecular links between ligand-induced activation of EGFR and regulation of endocytic trafficking of EGFR by HDAC6 are still not well-defined.

Na,K-ATPase, a P-type ATPase consisting of two essential subunits, the catalytic α-subunit and a β-subunit, and an auxiliary third subunit of the family of FXYD proteins, maintains intracellular ion homeostasis by pumping three Na ions out of and two K ions into the cell per cycle using the energy from ATP hydrolysis. The electrochemical gradient generated across the membrane by the pump activity of the enzyme is essential for many physiological processes, including nerve impulse transmission, nutrient uptake and electrolyte homeostasis. Recent studies revealed additional roles for Na,K-ATPase. For instance, it functions as a receptor for cardiotonic steroids activating intracellular signaling cascades and as a signaling scaffold [8–10]. The tyrosine kinase Src is the best characterized pump-associated signaling molecule, but Na,K-ATPase interacts with many other partners, including phosphoinositide 3-kinase (PI3K), caveolin-1, and EGFR [11–13]. We recently showed that ouabain inhibits EGF-induced actin stress fiber formation and cell motility in medulloblastoma cells [14]. Interestingly, Na,K-ATPase also binds to and is regulated by acetylated tubulin and may function as an anchorage site for microtubules [15]. On the other hand, it has been shown that EGF can trigger sodium influx upon receptor binding [16, 17]. We now suggest a novel link between EGFR, sodium influx, microtubule acetylation, and EGFR trafficking and provide evidence that activation of EGFR signaling by its ligand EGF induces a sodium influx thereby increasing tubulin acetylation. The increase in tubulin acetylation mediated by the inactivation of HDAC6 modulates trafficking of EGFR-containing vesicles. Consistent with this, blocking sodium influx in EGF-treated cells diminishes tubulin acetylation and delays EGFR degradation. Thus, these studies provide a novel regulatory mechanism in EGFR trafficking by sodium influx and the ion pump Na,K-ATPase.

## Results

### Sodium influx induces tubulin acetylation

**Fig. 1:**
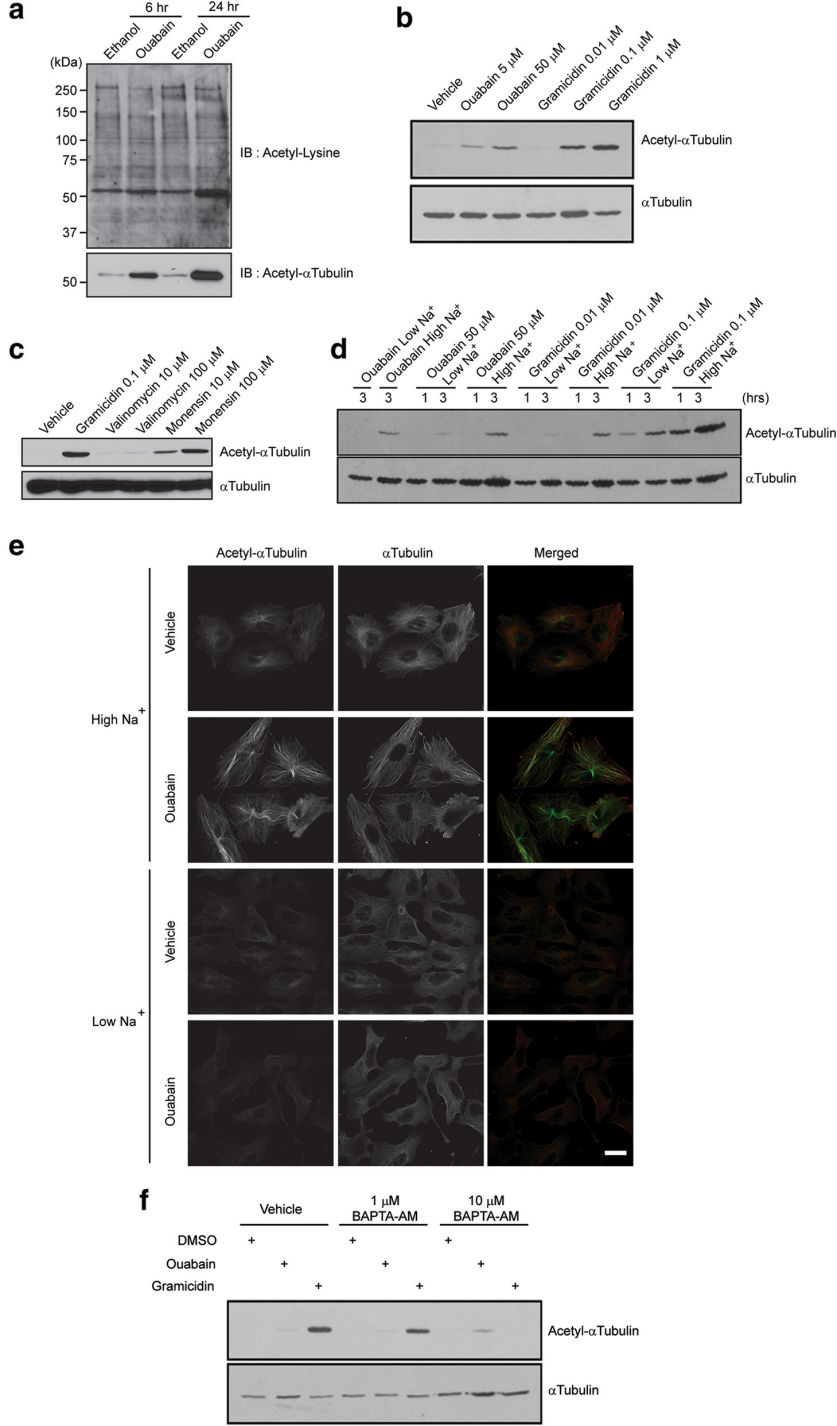
Sodium influx induces an accumulation of acetylated tubulin. **a** DAOY cells were incubated with 50 μM ouabain or ethanol (vehicle) for indicated times. Equal amounts of protein were separated by SDS-PAGE and immunoblotted with antibodies recognizing acetylated lysines (upper panel) or acetylated α-tubulin (lower panel). **b** Immunoblot for acetylated α-tubulin of DAOY cells treated for three hours at indicated concentrations with the sodium ionophore gramicidin A or the Na,K-ATPase inhibitor ouabain. An immunoblot for total α-tubulin ensured equal loading. **c** DAOY cells were treated for three hours at indicated concentrations with the potassium ionophore valinomycin or the sodium ionophore monensin. Gramicidin A was included for comparison. Equal amounts of protein were used for immunoblotting using antibodies against acetylated α-tubulin. An immunoblot for total α-tubulin ensured equal loading. **d** DAOY cells were treated with ouabain or gramicidin A as indicated in isotonic, sodium containing buffer (High Na^+^) or in a low-sodium buffer in which sodium was substituted with rubidium (Low Na^+^). Cells were lysed one or three hours after treatment and equal amounts of protein were immunoblotted for total and acetylated α-tubulin. **e** DAOY cells were incubated for three hours with 50 μM ouabain or vehicle in High Na^+^ or in Low Na^+^ buffer. The cells were fixed and immunostained with acetylated α-tubulin and pan-α-tubulin antibodies. For easier comparison, parameters for image acquisition were kept constant between samples. Scale bar, 10 μm. **f** DAOY cells were pre-incubated with the intracellular calcium chelator BAPTA-AM and then ouabain or gramicidin A were added for three hours. Equal amounts of protein were immunoblotted for acetylated α-tubulin. An immunoblot for total α-tubulin ensured equal loading.

Although it is now well-documented that acetylated tubulin associates with Na,K-ATPase to inhibit its pump function [18], less is known about the effects of reduced Na,K-ATPase function on tubulin acetylation. To test whether specific inhibition of Na,K-ATPase pump activity modulates the acetylation of tubulin, human DAOY medulloblastoma cells were treated with the cardiac glycoside ouabain. While ouabain specifically binds to Na,K-ATPase and inhibits its function, the ouabain concentrations and duration of treatment used in these experiments does not affect cell viability of DAOY cells [14]. First, to examine the acetylation pattern of whole cell lysates, immunoblotting was performed with an anti-acetylated lysine antibody that specifically recognizes acetylated lysine in a wide range of sequence contexts including acetylated histones, p53 and tubulin. We found little differences in the overall acetylation pattern between control and ouabain-treated cells, except for a noticeable band around 54 kDa that was increased in ouabain-treated cells (Fig. 1a, upper panel). From the size and abundance, we predicted that this band corresponded to acetylated tubulin. Further analyses by immunblotting and immunofluorescence with an anti-acetyl-α-tubulin antibody confirmed the presence of acetylated tubulin in ouabain-treated cells (Fig. 1a, lower panel, and Fig. 1e, upper panels). Since ouabain blocks the Na,K-ATPase activity leading to an increased intracellular sodium level, we next tested the effect of the mechanically distinct sodium ionophores, gramicidin A and monensin, on tubulin acetylation. Gramicidin A is a polypeptide obtained from *Bacillus brevis* that forms a β − helix inside the hydrophobic interior of the lipid bilayer thereby increasing the permeability of the cell membrane to monovalent cations such as sodium ions [19]. Monensin is a polyether antibiotic that forms a complex with sodium ions to permit these ions to travel across the lipid bilayer [20]. Both gramicidin A (Fig. 1b) and monensin (Fig. 1c) increased the acetylation of tubulin. In contrast, treatment with the potassium ionophore valinomycin did not change the level of acetylated tubulin (Fig. 1c). When NaCl was replaced by KCl the ability of ouabain or gramicidin to induce tubulin acetylation was greatly diminished. (Additional file 1: Figure S1A). Nevertheless, KCl treatment somewhat increased tubulin acetylation but not to the extend observed in ouabain or gramicidin-treated cells. Together these data suggest that an increase in intracellular sodium could result in increased tubulin acetylation. However, we cannot exclude that depolarization of the plasma membrane potential may contribute to the increase in tubulin acetylation observed in ouabain and gramicidin-treated cells.

To further prove that sodium intrusion triggers ouabain- or gramicidin A-induced tubulin acetylation, we treated DAOY cells with the same drugs in the presence of a low-sodium (Low Na^+^) buffer in which sodium ions were replaced with rubidium ions [21]. In Low Na^+^ buffer, while ouabain did not induce tubulin acetylation, gramicidin A markedly reduced the acetylated tubulin level when compared to that in regular sodium-containing, isotonic (High Na^+^) buffer (Figs. 1d, e, Additional file 1: Figure S1B). Posttranslational modification of tubulin by ouabain or gramicidin A was limited to acetylation, since no discernable differences were found in detyrosinated or glutamylated tubulin when compared to control cells (Additional file 1: Figure S1C). To test if increased intracellular calcium as a consequence of sodium influx contributes to tubulin acetylation, the accumulation of intracellular calcium in DAOY cells was chelated using BAPTA-AM, thereby neutralizing its effects on activation of CaM kinase, Calcineurin, Protein Kinase C and Calpain, to name a few. High concentrations of BAPTA-AM blocked the acetylation of tubulin (Fig. 1f), suggesting a role for increased intracellular calcium in sodium-induced tubulin acetylation. To further test whether intracellular calcium accumulation by itself is sufficient to induce tubulin acetylation, DAOY cells were incubated with a calcium ionophore A23187 (Additional file 1: Figure S1D). Interestingly, A23187 failed to increase the levels of acetylated tubulin, indicating that calcium signaling is required but not sufficient to induce tubulin acetylation. Taken together, these data suggest that an increase in intracellular sodium levels triggers the accumulation of acetylated tubulin, which may be mediated in part by a concomitant increase in intracellular calcium levels.

### EGF induces sodium-dependent tubulin acetylation

EGF induces various signaling cascades that trigger diverse molecular events. Whether the EGF-induced sodium increase modulates the acetylation of tubulin has not been shown. To test whether EGF can increase the acetylated tubulin levels via the accumulation of cytoplasmic sodium, we first confirmed using the green fluorescent sodium ion indicator CoroNa Green that EGF induces an increase in intracellular sodium in DAOY cells. The cells were loaded with CoroNa Green and the fluorescence signal was monitored by time-lapse microscopy after EGF was added to the bath solution. To obtain clearly detectable sodium signals, we not only treated the cells with EGF but included ouabain in our assay to prevent the Na,K-ATPase from extruding the accumulated sodium. Under these conditions EGF clearly delayed the fluorescence recovery rate indicating that EGF treatment indeed increased intracellular sodium in DAOY cells (Additional file 1: Figure S2A).

**Fig. 2:**
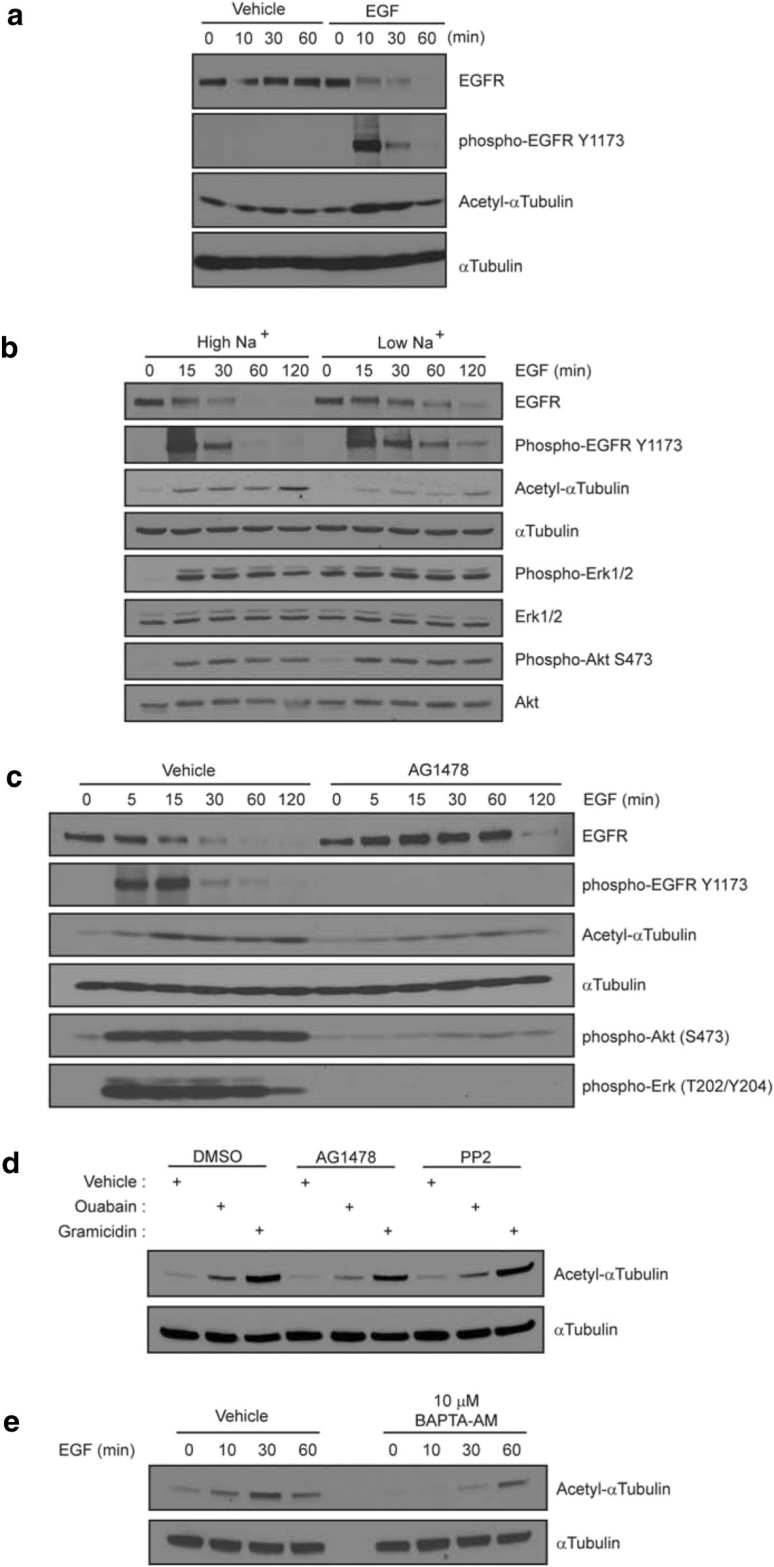
An EGF-induced increase in the intracellular sodium level results in increased acetylated tubulin levels. **a** DAOY cells were incubated with 10 ng/ml EGF or vehicle for indicated time points. Equal amounts of protein were immunoblotted for total and activated (Y1173) EGFR, and acetylated α-tubulin. An immunoblot for total α-tubulin was included to ensure equal loading. **b** DAOY cells were incubated for one hour in Low Na^+^ buffer and then treated with 10 ng/ml EGF for indicated times. Immunoblots were performed for total and activated (Y1173) EGFR, total and activated Erk1/2, total and activated (S473) Akt and acetylated α-tubulin. The total α-tubulin immunoblot confirmed equal loading. **c** DAOY cells were incubated for one hour with 10 μM AG1478 prior to addition of 10 ng/ml EGF. Immunoblots were performed as in **b**. **d** DAOY cells were incubated in the absence of EGF with either AG1478 or PP2 for one hour before 50 μM ouabain or 100 nM gramicidin A were added for an additional three hours. Equal amounts of protein were immunoblotted for acetylated and total α-tubulin. **e** DAOY cells were pre-incubated with BAPTA-AM and then 10 ng/ml EGF was added for indicated times. Equal amounts of protein were immunoblotted for acetylated α-tubulin. An immunoblot for total α-tubulin ensured equal loading.

EGF treatment not only revealed an increase in intracellular sodium but also a transient increase in acetylated tubulin as early as ten minutes upon addition of the ligand (Fig. 2a). To further test whether the EGF-induced sodium influx is sufficient to increase the level of acetylated tubulin, DAOY cells were treated with EGF in Low Na^+^ buffer. The EGF-induced increase in acetylated tubulin in Low Na^+^ was not as drastic as the one observed in High Na^+^, suggesting that EGF induces an accumulation of acetylated tubulin in a sodium dependent manner (Fig. 2b). To ascertain that the EGF treatment in Low Na^+^ is biologically functional, immunoblotting was performed with anti-phospho-EGFR (Y1173) antibody and phospho-specific antibodies that recognize the activated forms of the downstream effectors Erk1/2 and Akt. In both High Na^+^ and Low Na^+^ buffers, EGF activated EGFR, Erk1/2 and Akt to a similar extend suggesting that activation of canonical EGFR signaling is independent of the EGF-induced sodium influx (Fig. 2b). Consistent with this, blockade of the MAPK signaling cascade with the MEK inhibitor PD98059 or Akt signaling with the PI3K inhibitor LY294002 did not significantly affect the accumulation of acetylated tubulin induced by ouabain or gramicidin A (Additional file 1: Figure S2B). However, the kinase activity of EGFR contributes to the accumulation of acetylated tubulin, since AG1478, a specific inhibitor of the EGFR kinase activity, reduced the EGF-induced increase in the levels of acetylated tubulin (Fig. 2c), suggesting that both EGFR tyrosine kinase dependent and independent mechanisms might be contributing to the accumulation of acetylated tubulin. AG1478 did not abolish the increase in tubulin acetylation observed in cells treated with ouabain or gramicidin A nor did the Src inhibitor PP2 (Fig. 2d). Together, these data suggest that accumulation of acetylated tubulin and activation of the well-described EGFR-MAPK and EGFR-Akt signaling cascades are mediated by independent mechanisms.

An increase in intracellular sodium is often accompanied by a calcium influx and as shown in Fig. 1f, calcium was required for the accumulation of acetylated tubulin in ouabain- or gramicidin A-treated cells. Similarly, in cells treated with EGF in the presence of BAPTA, chelation of intracellular calcium significantly delayed the accumulation of EGF-induced acetylated tubulin (Fig. 2e). To further dissect the downstream effectors of calcium signaling, DAOY cells were pretreated with either CaMK inhibitor (KN-62), calpain inhibitors (ALLN, Calpeptin), a calcineurin inhibitor (cyclosporin A; Additional file 1: Figure S3) or the PKC inhibitor (Bim1; data not shown) before incubation with either EGF, ouabain or gramicidin A. Surprisingly, none of these inhibitors seemed to have a significant effect on the level of acetylated tubulin and experiments are in progress to identify other calcium-dependent mediators of tubulin acetylation.

### Sodium influx attenuates EGFR trafficking through inhibition of HDAC6 activity and acetylation of tubulin

**Fig. 3:**
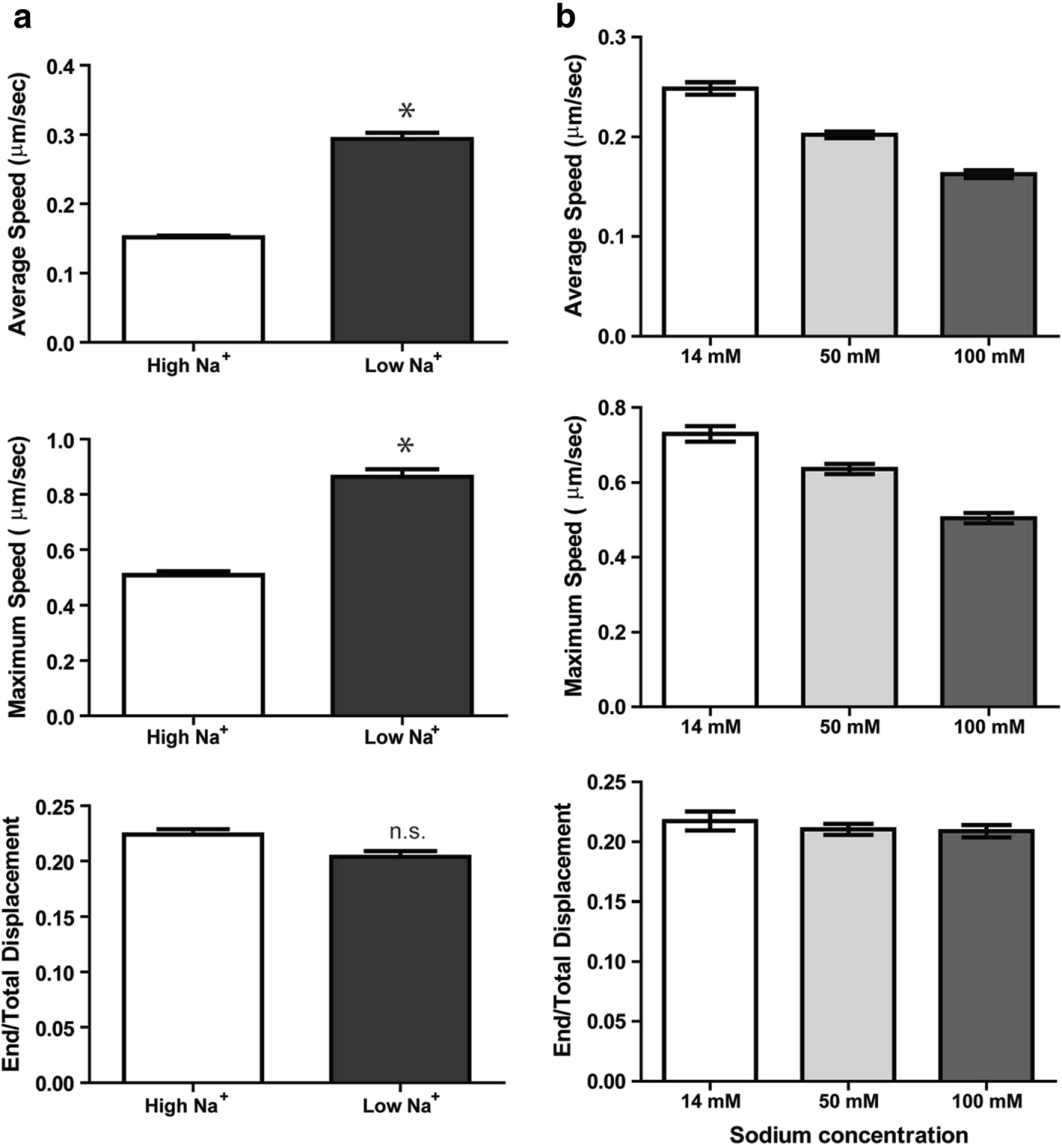
Intracellular sodium levels regulate vesicle trafficking. **a** DAOY cells were preincubated with either High Na^+^ or Low Na^+^ buffer for 30 minutes and then stimulated with 40 ng/ml of AlexaFluor 488-conjugated EGF for an additional 15 minutes during which time epifluorescence images were taken with a cooled CCD camera at a rate of one frame/second for a total of 900 frames. The motility of EGF-positive vesicles was analyzed using Slidebook software and average speed, maximum speed and end/total displacement were plotted. *, P < 0.0001. **b** EGF was added to DAOY cells incubated in buffers of increasing sodium concentrations and the motility of EGF-positive vesicles was analyzed as in A.

Ligand-induced EGFR signaling is downregulated through internalization and lysosomal degradation. Consistent with this, EGFR levels in DAOY cells gradually reduced following EGF treatment in High Na^+^ (Fig. 2a) but in Low Na^+^, the decrease not only in total EGFR protein but also in the levels of activated phospho-EGFR was clearly delayed (Fig. 2b). Therefore, we hypothesized that the EGF-induced sodium influx plays a role in terminating EGFR signaling through modulating EGFR internalization and degradation, with trafficking possibly controlled by altered levels of tubulin acetylation. We first treated DAOY cells with Alexa Fluor 488-conjugated EGF and monitored trafficking of EGF-positive vesicles by live cell imaging, comparing displacement, average and maximum speed of vesicles in High Na^+^ and Low Na^+^ buffers (Fig. 3a). The average speed and the maximum speed of EGF-positive vesicles were significantly higher in cells incubated in Low Na^+^ buffer, but the movement pattern as determined by the ratio of end versus total displacement was not different in the presence or absence of sodium (Fig. 3a). The increased vesicle speed in Low Na^+^ buffer was significantly reduced by treatment of nocodazole that disrupts microtubules suggesting that even in Low Na^+^ buffer the rapid vesicle movement still requires mircotubules (Additional file 1: Figure S4). Furthermore, the average and maximum speed of vesicles decreased with increasing concentrations of Na^+^ in the buffer (Fig. 3b), supporting our notion that vesicle trafficking may be modulated by sodium levels.

**Fig. 4:**
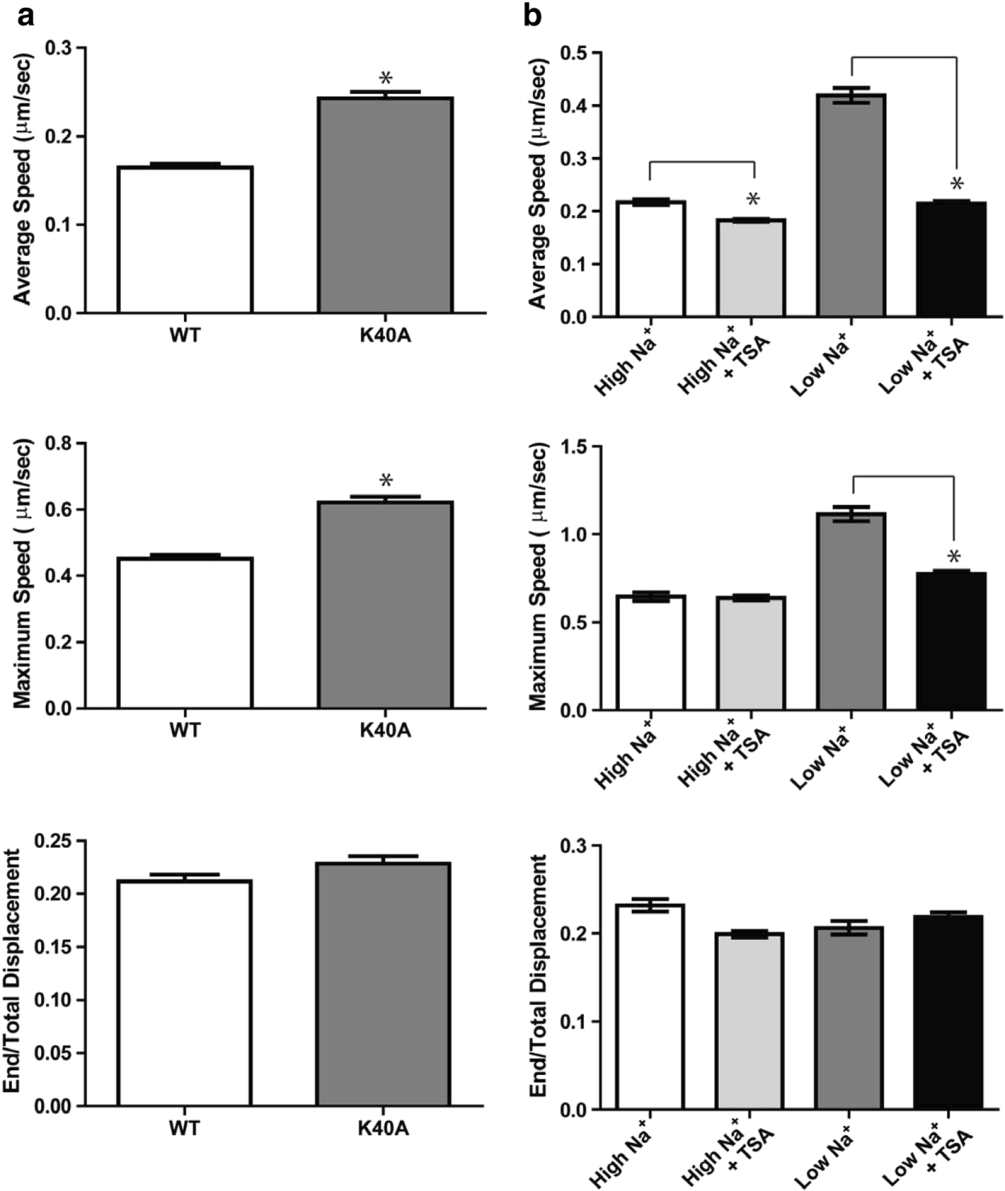
Increased acetylated tubulin decreases the motility of EGF-positive vesicles. **a** DAOY cells were transiently transfected with either wild-type tubulin (WT) or a nonacetylated mutant of tubulin (K40A). AlexaFluor 488-EGF was added in High Na^+^ buffer and images were acquired as described above. Average speed, maximum speed and the end/total displacement are shown. *, P < 0.001. **b** DAOY cells were preincubated with 100 nM TSA for 15 minutes and then AlexaFluor 488-EGF was added. Vesicles were tracked as described in figure legend 3. *, P < 0.001.

To test whether the sodium-dependent change in the level of acetylated tubulin mediates the increase in the speed of EGF-positive vesicles, we first inhibited tubulin acetylation using a dominant negative mutant of α-tubulin in which the predominately acetylated lysine is mutated to alanine (K40A) [22]. The K40A tubulin mutant was transiently expressed in DAOY cells and the trafficking pattern of the EGF-positive vesicles was determined. As compared to control cells, expression of the K40A tubulin mutant resulted in a statistically significant increase in the average and the maximum speed of EGF-positive vesicles but did not significantly affect the end/total displacement (Fig. 4a). On the contrary, increasing the level of acetylated tubulin by inhibition of HDAC6 with the pan-HDAC inhibitor trichostatin A (TSA) almost completely abolished the increase in vesicle speed observed in Low Na^+^ and the levels were similar to that of control cells in High Na^+^ (Fig. 4b). TSA had a minimal effect on the speed of EGF-positive vesicles in High Na^+^ and, as expected, did not significantly affect end/total displacement in both Low Na^+^ and High Na^+^ (Fig. 4b). Taken together, these data strongly support our hypothesis that trafficking of EGF-containing vesicles is precisely regulated by altering the acetylation status of microtubules through regulating HDAC activity in a sodium-dependent manner.

### Sodium influx inhibits HDAC6 to increase the levels of acetylated tubulin

**Fig. 5:**
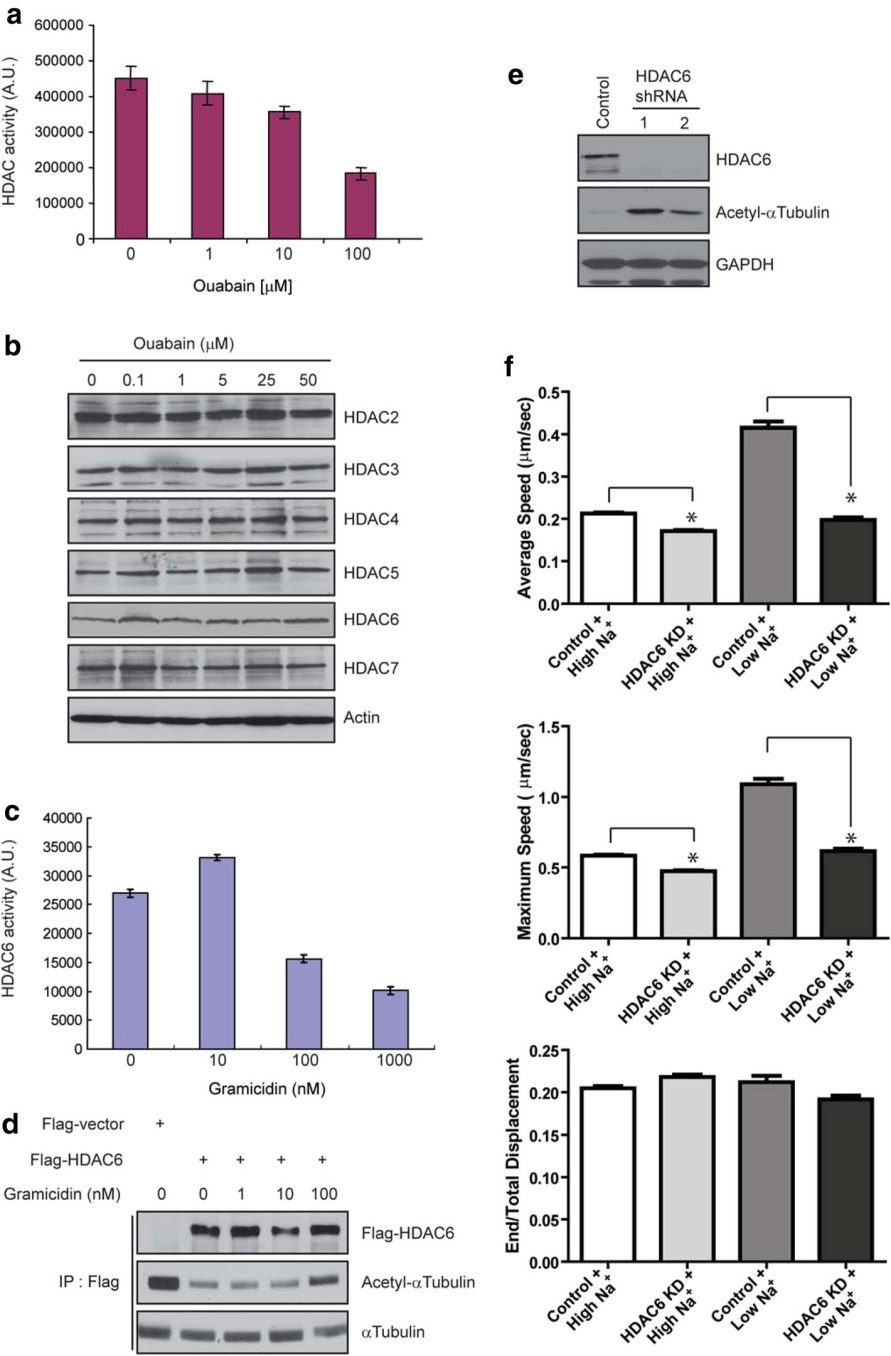
Increased intracellular sodium inhibits HDAC6 activity. **a** DAOY cells were incubated with increasing concentrations of ouabain for three hours and then lysed for an *in vitro* HDAC activity assay as described in Materials and Methods. **b** DAOY cells were incubated with indicated concentrations of ouabain and the protein levels of the indicated HDAC isoforms were determined by immunoblotting. Actin immunoblot ensures equal loading. **c** After incubation with indicated concentrations of gramicidin A, HDAC6 was immunoprecipitated with a specific antibody and then subjected to an *in vitro* HDAC activity assay using a fluorescent substrate. **d** DAOY cells were transiently transfected with Flag-HDAC6 cDNA. The cells were incubated with the indicated concentrations of gramicidin A followed by immunoprecipitation of HDAC6. The immunoprecipitates were washed extensively and then incubated with polymerized microtubules to measure deacetylase activity. The reaction was terminated by adding lysis buffer and the samples were processed for immunoblotting with indicated antibodies. **e** Immunoblot for HDAC6 of stable clones of DAOY cells expressing HDAC6 shRNA. Control cells were transfected with vector only and processed in parallel. A GAPDH blot ensures that equal amounts of protein were used for analysis. **f** The HDAC6 knockdown cells were incubated with either High Na^+^ or Low Na^+^ buffer and the trafficking patterns of EGF-positive vesicles were analyzed as described in figure legend 3. *, P < 0.0001.

Given that both ouabain and gramicidin A induced the accumulation of acetylated tubulin through increasing intracellular sodium levels (Fig. 1) and that the speed of EGF-positive vesicle trafficking is regulated by the level of acetylated tubulin through regulating the activity of HDACs (Figs. 3 and 4), we hypothesized that intracellular sodium may regulate HDAC activity. Indeed, treatment of DAOY cells with increasing concentrations of ouabain resulted in a decrease of the total HDAC activity (Fig. 5a). This decrease was not accompanied by noticeable changes in the protein levels of various HDAC isoforms, including HDACs 2–5 and HDAC7 (Fig. 5b).

Since HDAC6 is the major HDAC isoform regulating the level of acetylated tubulin we tested if gramicidin A treatment affects HDAC6 activity. Similar to the effect of ouabain on total HDAC activity (Fig. 5a), we observed a dose-dependent reduction in HDAC6 activity in gramicidin A-treated cells (Fig. 5c). To validate these data, we performed an *in vitro* tubulin deacetylation assay. In this assay, cells transiently expressing Flag-tagged HDAC6 were treated with different concentrations of gramicidin A and then HDAC6 was immunoprecipitated. The precipitates were incubated with *in vitro* polymerized microtubules, the reaction was stopped and the levels of acetylated tubulin were determined by immunoblotting. HDAC6 precipitated from cells treated with 100 nM gramicidin A showed a lower deacetylation activity than HDAC6 precipitated from control cells or cells treated with 1 or 10 nM of gramicidin A (Fig. 5d). Both ouabain and gramicidin A treatment also increased the acetylation of cortactin, another HDAC6 substrate (data not shown).

To directly address whether HDAC6 plays a role in regulating the trafficking of EGF-containing vesicles following EGF treatment and concomitant sodium increase, HDAC6 shRNA was stably expressed in DAOY cells to knockdown the protein (Fig. 5e). In both of the two independent clones selected, knockdown of HDAC6 was accompanied by an increase in the levels of acetylated tubulin when compared to that with control shRNA (Fig. 5e). Further, both the average and maximum speeds of EGF-positive vesicles following EGF treatment were significantly reduced by HDAC6 knockdown both in High Na^+^ and Low Na^+^ buffers (Fig. 5f). Taken together, these data support our hypothesis that an EGF-induced increase in intracellular sodium level inhibits HDAC6 activity, resulting in increased tubulin acetylation, and regulation of EGFR trafficking.

### EGF regulates vesicle trafficking between endosomes via sodium-dependent regulation of HDAC6 activity

**Fig. 6:**
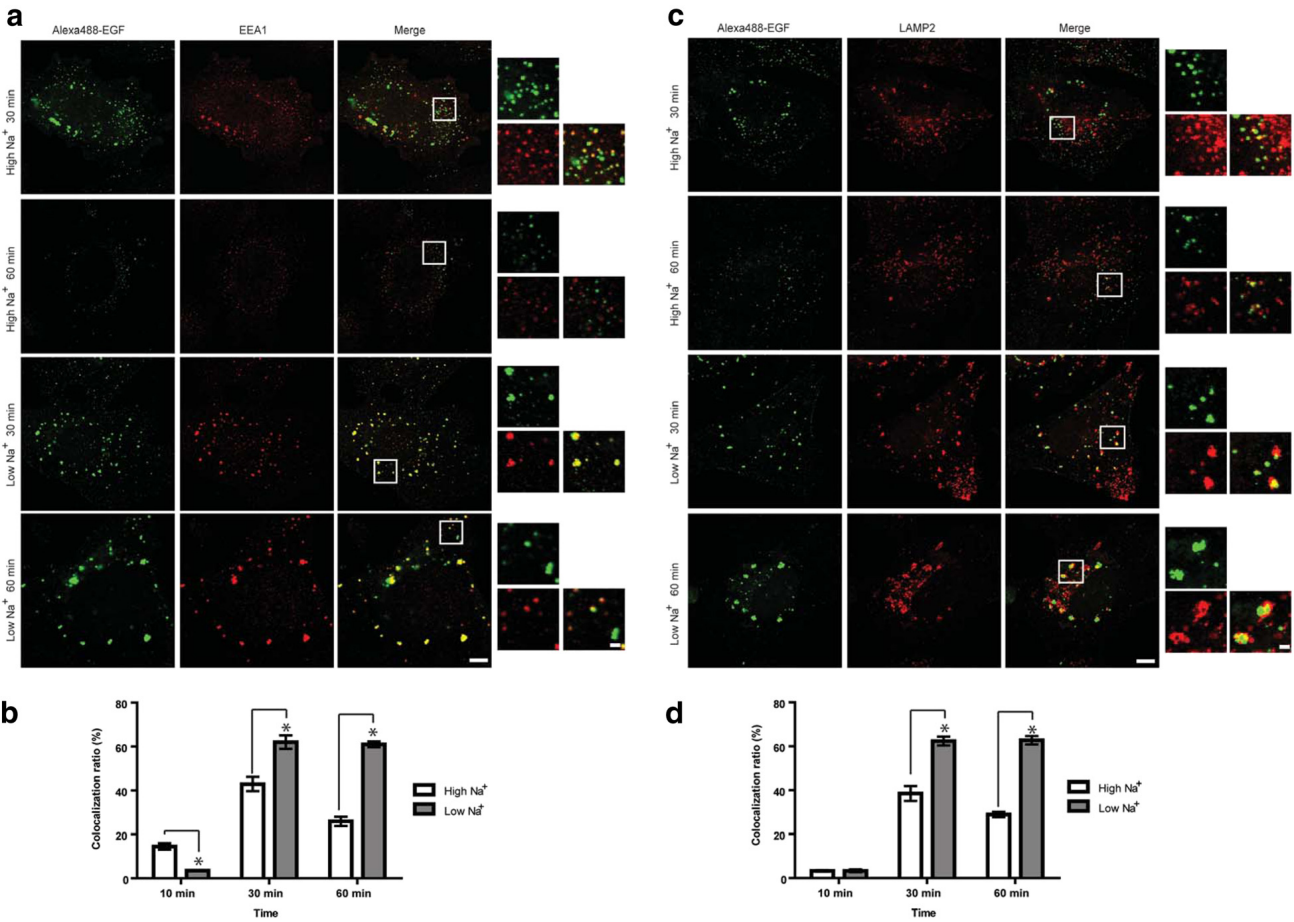
Acetylated tubulin mediated by intracellular sodium level regulates vesicle trafficking between endosomes. **a** DAOY cells were treated with AlexaFluor 488-EGF for 30 minutes **a**, **b**) or 60 minutes (C, D) in High Na^+^ or Low Na^+^ buffer. Immunofluorescence of early endosomes (EEA1) (A) and late endosomes (LAMP2) **c** are shown. The squares depict the areas of higher magnification. Scale bars represent 10 μm (low magnification) and 1 μm (high magnification). (B and D) The colocalization ratio was calculated using ImageJ and plotted as mean with standard errors.

EGF binding to its receptor not only activates diverse signaling pathways, but formation of the EGF-EGFR complex also triggers clathrin-dependent endocytosis. The vesicles that are being formed fuse with early endosomes and then, to terminate signaling, the EGF-EGFR complex transits to late endosomes and ultimately to lysosomes for degradation. During this journey, vesicles move along microtubule tracks and the (de)acetylation of tubulin is one of the key determinants regulating this trafficking [23]. Given that EGF treatment induces sodium influx to reduce HDAC6 activity followed by an increase in acetylated tubulin levels and a decrease in the speed of vesicle trafficking, we hypothesized that this change in vesicle trafficking speed modulates the rate of transition from either early to late endosomes or late endosomes to lysosomes. Thus, we analyzed the intracellular localization of EGF-positive vesicles in DAOY cells in High Na^+^ or Low Na^+^ buffer by co-localization with compartment-specific markers, EEA1 and LAMP2, for early and late endosomes, respectively. Ten minutes after treatment, the majority of EGF-positive vesicles localized to the periphery of the cells with a minor population co-localizing with EEA1-positive early endosomes at the perinuclear region (Fig. 6a, b). This co-localization peaked at 30 minutes and slowly subsided by 60 minutes in High Na^+^ buffer. In contrast, at 10 min after treatment only a very small subpopulation of EGF-positive vesicles localized to early endosomes in Low Na^+^ buffer. However, the co-localization ratio in Low Na^+^ buffer exceeded that in High Na^+^ by 30 minutes after treatment and this ratio did not decline even after 60 minutes, suggesting that modulation of acetylated tubulin levels by different intracellular sodium levels may regulate the turnover of EGF-EGFR complexes through controlling the vesicle trafficking rate. Complementarily, only a small fraction of EGF-positive vesicles localized to LAMP2-positive late endosomes after 10 minutes of treatment in both High Na^+^ and Low Na^+^ buffers (Fig. 6c, d). However, at 30 minutes, more EGF-positive late endosomes accumulated in Low Na^+^ buffer and persisted up to 60 minutes of treatment (Fig. 6c, d). Interestingly, we observed that in Low Na^+^ buffer the vesicles seemed to aggregate forming multivesicular structures rather than fusing to form a larger vesicle. Studies are in progress to test whether the failure of fusion may hamper the transition from late endosomes to lysosomes thereby delaying the degradation of EGF-EGFR complexes.

## Discussion

**Fig. 7:**
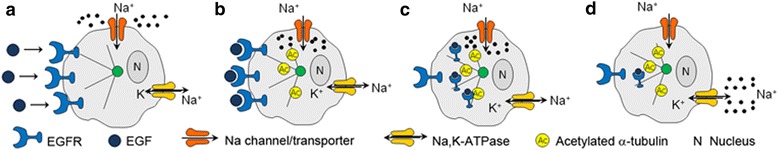
Schematic model. **a** Resting cell in the absence of ligand. **b** Ligand-binding leading to increased intracellular sodium and increased tubulin acetylation (mediated through inhibition of HDAC6). **c** The ligand-receptor complex is internalized and trafficked for degradation. **d** The ligand-receptor complex is degraded and signaling is terminated. Na,K-ATPase restores low intracellular sodium levels.

Generally, activation of EGFR signaling is terminated by endocytosis, and targeting of the receptor complex to lysosomes for degradation relies on transport of the cargo vesicles along microtubule tracks. Recent studies show that acetylation of α-tubulin regulates intracellular cargo transport [5, 22, 23] including transport of EGFR-containing vesicles [7, 24]. We now show that sodium influx induces tubulin acetylation via suppression of HDAC6 activity to modulate EGFR trafficking. Moreover, EGF-binding to its receptor provokes sodium influx and increases tubulin acetylation to modulate the speed of EGF-containing vesicles, downregulate EGFR levels and terminate EGFR signaling. Two sets of experiments indeed support the hypothesis that HDAC6-induced tubulin deacetylation can regulate EGFR trafficking. (1) Mutation of the lysine residue (K40) to alanine resulted in an increased speed of EGF-positive vesicles, suggesting that acetylation of this residue can modulate movement of EGF-positive vehicles along microtubules. (2) Knockdown of HDAC6 led to a global increase of acetylation on the same K40 residue resulting in altered trafficking of EGF-positive vesicles even in Low Na^+^ conditions. Together these data support our conclusion that HDAC6-incuded tubulin deacetylation on the lysine 40 residue can directly influence EGF vesicle motility. While at this point we cannot completely exclude membrane depolarization to be a contributing factor, we suggest that an increase in intracellular sodium is a component of an auto-regulatory loop that regulates termination of EGFR signaling through trafficking of the EGF-EGFR complex and includes HDAC6 and tubulin acetylation (Fig. 7).

Under physiological conditions, the intracellular sodium concentration is closely regulated to maintain the electrochemical gradient that is central to transmembrane transport. It is also tightly coupled to homeostasis of other ions such as calcium or pH. In addition, recent studies suggest that transient increases in the intracellular sodium level have an active role in signaling contributing to several cellular functions including cell migration and proliferation [25]. While EGF can trigger sodium influx upon receptor binding [16, 17], its physiological relevance is not clear. We recently showed that ouabain inhibits EGF-induced Erk1/2 and Akt activation and prevents EGF-induced formation of actin stress fibers and cell motility in medulloblastoma cells [14]. Since Na,K-ATPase functions as a signaling scaffold it is possible that Na,K-ATPase might act as a checkpoint to integrate EGF-associated signaling pathways.

The EGF-induced increase in intracellular sodium can promote the uptake of calcium via Na^+^/Ca^2+^ exchange [26]. Indeed, in our experiments, the calcium chelator BAPTA-AM significantly delayed EGF-induced tubulin acetylation (Fig. 2e) and blocked the acetylation of tubulin induced by ouabain or gramicidin A (Fig.1f). It is well-known that EGF receptor signaling can also activate phospholipase Cγ to elevate the intracellular calcium level and activate PKC. Nevertheless, the PKC inhibitor Bim1 or inhibitors for other commonly known downstream effectors of calcium signaling such as CaMK (KN-62), Calpain (ALLN, calpeptin) and Calcineurin (cyclosporine A) had no significant effect on EGF, ouabain or gramicidin A-induced tubulin acetylation, suggesting that they are not key players in the EGF-HDAC6-tubulin acetylation pathway. In addition, the calcium ionophore A23187 by itself did not induce tubulin acetylation and thus, while calcium influx may be required it is not sufficient to induce tubulin acetylation. In various cell types EGF can increase the intracellular pH by augmenting the Na^+^/H^+^ antiport activity [16]. In our cells, inhibiting the Na^+^/H^+^ antiport activity with amiloride did not significantly reduce the acetylation of tubulin (data not shown) suggesting that Na,K-ATPase itself may mediate EGF-induced tubulin acetylation. Together, our studies are consistent with Na,K-ATPase being an integral part of an auto-regulatory loop that regulates EGFR trafficking through HDAC6 and tubulin acetylation in order to terminate EGF-induced signaling. The intracellular sodium concentration is mainly set and governed by the activity of the Na,K-ATPase. Interestingly, Na,K-ATPase also functions as a signaling scaffold [8–10], transactivates EGFR [13], and binds to and is regulated by acetylated tubulin [15, 18]. Experiments are in progress to identify whether Na,K-ATPase is a key signaling molecule that regulates the sodium signal to inhibit HDAC6 activity.

In contrast to class I HDACs that are primarily localized to the nucleus, HDAC6 can shuttle between the cytosol and the nucleus and sodium may possibly regulate only one of these HDAC6 subsets. With the HDAC inhibitors TSA and tubastatin A being much more effective to induce tubulin acetylation as compared to ouabain or gramicidin A, it is also possible that sodium regulates only spatially restricted HDAC6, either in close proximity to the plasma membrane or microtubule networks. This would be consistent with our observation that HDAC6 inactivation by ouabain or gramicidin A was not as prominent as that with HDAC inhibitors. Acetylated tubulin is known to inhibit the pump activity of Na,K-ATPase through interaction with the fifth cytoplasmic domain of the α-subunit, which could act as a positive feedback to locally maintain a high intracellular sodium level by reducing the pump activity. Functional interactions between Na,K-ATPase and EGFR are well-documented and it is tempting to speculate that EGF-induced sodium influx increases tubulin acetylation through a local inactivation of HDAC6 allowing for a spatial fine tuning of EGFR internalization and signaling. While we found a higher rate of receptor down-regulation in High Na^+^ buffer we initially expected a higher motility of EGF-positive vesicles in High Na^+^ buffer compared to Low Na^+^ solution. Nevertheless, we found that the speed of the vesicles was accelerated in Low Na^+^ buffer. It is possible that the higher and more erratic motility of EGF-positive vesicles in Low Na^+^ conditions resulted in a less efficient delivery into late endosomes/lysosomes for actual protein degradation to occur. This is consistent with our observation that in Low Na^+^ buffer the vesicles seemed to aggregate forming multivesicular structures rather than fusing to form a larger vesicle resembling lysosomes. Studies are in progress to test whether the failure of fusion may hamper the transition from late endosomes to lysosomes thereby delaying the degradation of EGF-EGFR complexes.

A previous report shows that EGF can downregulate HDAC6 activity through phosphorylation of HDAC6 at tyrosine 570 [7]. While pretreatment of DAOY cells with the EGFR kinase inhibitor AG1478 prevented EGF-induced tubulin acetylation, we were not able to detect tyrosine phosphorylated HDAC6 after EGF treatment in DAOY cells (data not shown). Recently, other kinases that phosphorylate HDAC6 at various sites and regulate the enzyme’s activity towards α-tubulin have emerged. For example, the centrosomal Aurora A kinase that regulates mitotic entry phosphorylates HDAC6 to promote ciliary disassembly by enhancing the ability of HDAC6 to deacetylate acetylated tubulin [27]. The G-protein-coupled receptor kinase 2 stimulates HDAC6 activity through phosphorylation as well [28]. PKCα enhances HDAC6 activity [29] but in our studies the PKC inhibitor Bim1 did not prevent the increase in tubulin acetylation induced by ouabain or gramicidin A (data not shown). Erk1/2 phosphorylates HDAC6 at serine 1035 and threonine 1031, and EGF increases HDAC6 phosphorylation at serine 1035 [30]. Both ouabain and gramicidin A are known to activate Erk1/2, however PD98059 did not affect tubulin acetylation in ouabain and gramicidin A-treated cells. While we were able to show that an increase in the intracellular sodium level leads to increased levels of acetylated tubulin through HDAC6 inhibition, the exact mechanisms that regulate HDAC6 activity under these conditions remain elusive. And since EGF not only activates EGFR but also sodium influx, our data do not exclude that both direct phosphorylation of HDAC6 by EGFR, and sodium influx-induced inactivation may contribute to the increase in tubulin acetylation upon activation of EGFR. Moreover, glycogen synthase kinase (GSK) 3β phosphorylates HDAC6 at serine 22 and enhances HDAC6 deacetylase activity toward α-tubulin [31]. It is well established that cardiac glycosides activate Akt, an upstream regulator of GSK3β. Ιndeed, in DAOY cells both ouabain and gramicidin A induced Akt phosphorylation (Additional file 1: Figure S2B), nevertheless the Akt inhibitor LY294002 did not prevent the associated increase in tubulin acetylation. However replacement of sodium with lithium, a direct inhibitor of GSK3β, resulted in accumulation of acetylated tubulin (data not shown). Thus, GSK3β may be a candidate molecule that mediates sodium-induced inhibition of HDAC6 and studies are in progress to delineate the significance of this pathway.

## Conclusion

Despite the complex nature of EGFR signaling, targeted therapeutic approaches have become an intense focus in drug development for various cancers. Currently, the two major clinically successful strategies are tyrosine kinase inhibitors and monoclonal anti-receptor antibodies. Tyrosine kinase inhibitors bind the magnesium-ATP-binding pocket of the tyrosine kinase domain to block ligand-induced receptor autophosphorylation, thereby disrupting tyrosine kinase activity and downstream signaling. Therapeutic anti-EGFR antibodies aim at inhibiting ligand binding, accelerating receptor internalization and degradation in response to antibody-directed clustering and aggregation, and induction of antibody-dependent cell-mediated cytotoxicity. Despite initial therapeutic response, EGFR inhibitors are prone to intrinsic and acquired resistance and EGFR mutations. Moreover, altered cellular localization, impaired protein trafficking, and degradation leading to increased receptor levels are known to contribute to therapeutic resistance [32]. For example, acquired resistance to the anti-EGFR antibody Cetuximab has been associated with altered EGFR ubiquitination, leading to dysregulation of EGFR internalization and degradation, and sustained signaling [33, 34]. We now show that intracellular sodium regulates trafficking of the EGFR receptor and we suggest that changes in the intracellular sodium level in tumor cells may contribute to therapeutic resistance to therapeutic anti-EGFR antibodies. Furthermore, it is possible that changes in Na,K-ATPase expression or function as the principal regulator of intracellular sodium homeostasis may contribute to resistance to targeted EGFR inhibitors. Studies are in progress to determine whether intracellular sodium or Na,K-ATPase levels have clinical implications for the therapy with targeted EGFR inhibitors.

## Methods

### Reagents and cell culture

Ouabain, gramicidin A, valinomycin, monensin, PP2, PD98059, LY294002 and BAPTA-AM were obtained from Sigma (St. Louis, MO) and EGF was from Invitrogen. Isotonic sodium buffer was composed of 140 mM NaCl, 3 mM KCl, 1 mM MgCl_2_, 1 mM CaCl_2_, 5 mM Glucose and 20 mM HEPES, pH 7.4. For low-sodium buffer NaCl was replaced with RbCl [21]. Antibodies recognizing EGFR, phospho-EGFR (Y1173), acetyl-α-tubulin (K40), α-tubulin, phospho-Akt (S473), phospho-Erk (T202/Y204), GAPDH, HDAC6, Flag (M2), and EEA1, were purchased from Cell Signaling (Beverly, MA), anti-LAMP2 was obtained from Developmental Studies Hybridoma Bank, University of Iowa.

Flag-tagged HDAC6 cDNA was obtained from Addgene (Cambridge, MA) and transfected into human medulloblastoma DAOY cells (ATCC, Rockville, MD) using Lipofectamine 2000 (Invitrogen). For stable knockdown, shRNA targeting human HDAC6 was inserted into pSUPER.basic (Oligoengine, Seattle, WA). Stable clones were selected after treatment with G418. Control clones were obtained in parallel after transfection with pSUPER vector.

### Immunoblotting

Immunoblots were performed as described [14]. Briefly, cell lysates were prepared in a buffer containing 20 mM Tris (pH 7.5), 150 mM NaCl, 1 mM EDTA, 1 mM EGTA, 1% Triton X-100, 2.5 mM sodium pyrophosphate, 1 mM β-glycerolphosphate, 1 mM sodium vanadate, 1mM phenylmethylsulfonyl fluoride and a cocktail of protease inhibitors (5μg/ml of antipapain, leupeptin and pepstatin, each). 20–40 μg of cell lysate was separated by 8% SDS-PAGE, transferred to nitrocellulose membrane and blocked in 5% non-fat milk in Tris-buffered saline with 0.1% Tween 20. The blocked membranes were incubated with primary antibodies overnight at 4°C. Protein bands were detected with HRP-conjugated secondary antibodies and Enhanced Chemiluminescense Plus (GE Healthcare, Piscataway, NJ). Representative immunoblots from at least three independent experiments are shown.

### HDAC activity and in vitro tubulin deacetylation assay

HDAC activity was measured in total cell lysates or after immunoprecipitation with isoform-specific antibodies using the fluorometric HDAC Activity Assay kit (Abcam, Cambridge, MA) as previously described [35]. Briefly, cell lysates or immunoprecipitates were incubated with assay buffer containing 0.2 mM Boc-Lys(Ac)-AMC as a substrate for 30–60 min. Subsequently, trypsin was added with 1 μM trichostatin A (TSA) to terminate the deacetylation and cleave deacetylated substrates. Fluorescence was measured with excitation at 390 nm and emission at 460 nm.

For the tubulin deacetylation assay, cells transiently expressing Flag-tagged HDAC6 were lysed in a buffer of 50 mM Tris–HCl, pH 7.6, 120 mM NaCl, 0.5 mM EDTA, 0.5% NP-40 with protease inhibitors and tagged HDAC6 was immunoprecipitated with anti-Flag (M2) antibody. After several washes in assay buffer (10 mM Tris–HCl, pH 8.0, 10 mM NaCl), immunoprecipitated proteins were incubated with 10 μg of microtubule associated protein (MAP)-stabilized microtubules [polymerized from a MAP-rich tubulin fraction (Cytoskeleton, Inc)] for two hours at 37°C. Supernatant and beads were separated by centrifugation and subjected to immunoblotting as described [36].

### Immunofluorescence microscopy

Immunostaining was performed as previously described [36]. Cells were imaged under a Leica SP5 (Buffalo Grove, IL) confocal microscope with a x63 oil immersion objective using the 488, 543, 633 nm laser lines for excitation for sequential image acquisition. For EGF treatment, cells were serum-starved overnight and then EGF-AlexaFluor 488 (40 ng/ml) (Life Technologies, Grand Island, NY) was added for indicated time points. Quantification of the colocalization between EGF-AlexaFluor 488 and the endosomal marker proteins was performed as described by Vanlandingham et al. [37]. Briefly, randomly chosen fields of cells (5–10 cells/field) were selected and analyzed with ImageJ software and the Colocalization plug-in (Pierre Bourdoncle, Institut Jacques Monod, Service Imagerie, Paris). Binary images from each channel by automated channel thresholding were used to generate a colocalized image with the "min" operation of the Image calculator function. Areas of the positive pixels from the colocalized image and the binary image of EGF-AlexaFluor 488 were measured, respectively. Data were plotted as the ratio of area from those two images. All data represent the average of three independent experiments with a total of 70–90 cells analyzed in total. Statistical analysis was performed by unpaired t-test between two groups (control and experimental). P values larger than 0.05 were regarded as statistically non-significant.

### Live cell imaging

Cells grown on glass-bottom dishes were serum-starved overnight and then incubated with EGF-AlexaFluor 488 (40 ng/ml). The dish was placed on a Zeiss Axio Observer Z1 (Peabody, MA) inverted microscope (x100 oil immersion objective) equipped with a CO_2_ and temperature-regulated environmental chamber. Time-series images were taken after 5 min of EGF treatment with an interval of one second for ten minutes. Images were acquired with a QuantEM:512SC electron-multiplying CCD camera (Photometrics, Tucson, AZ) and the motility of EGF-AlexaFluor 488 positive vesicles was analyzed from the time-lapse images using Slidebook software (Intelligent Imaging Innovations Inc., Denver, CO). In brief, binary masks were generated after background subtraction and then the individual objects (vesicles) were tracked/analyzed using the particle tracking functions of the software. After removing vesicles smaller than ten pixels in size, vesicles which stayed in the same focal plane for at least ten frames were used to analyze the movement. Differences in the speed of vesicles in two groups was assessed by unpaired t-test and P values larger than 0.05 were regarded as non-significant.

## Additional file

Additional file 1: Figure S1. Sodium-induced post-translational modifications of tubulin is limited to acetylation. Figure S2. EGF treatment increases intracellular sodium. Figure S3. Sodium-induced acetylation of tubulin and calcium signaling. Figure S4. Average vesicle speed in nocodazole-treated cells. (PDF 360 kb)

## Abbreviations

EGFR: epidermal growth factor receptor
HAT: histone acetyltransferase
HDAC: histone deacetylase
High: Na^+^ isotonic sodium-containing buffer
Low: Na^+^ low sodium buffer
PI3K: phosphoinositide 3-kinase
TBS: tris-buffered saline
