# Supplementary material for: EGF-induced sodium influx regulates EGFR trafficking through HDAC6 and tubulin acetylation
Source: BMC Cell Biol. 2015 Sep 18;16:24. doi: 10.1186/s12860-015-0070-8 (PMC4574528; doi:10.1186/s12860-015-0070-8)

SUPPLEMENTARY FIGURE LEGENDS

Supplementary Figure 1. Sodium-induced post-translational modifications of tubulin is limited to acetylation. (A) DAOY cells were pretreated for 30 minutes with a buffer in which NaCl was replaced with KCl before incubation with ouabain or gramicidin for an additional three hours. Immunoblots were performed as indicated. (B) DAOY cells were preincubated in isotonic sodium (High Na^+) or low–sodium (Low Na^+) buffer for one hour before adding vehicle or gramicidin A for an additional three hours. Immunostaining for total and for acetylated tubulin are shown. For easier comparison, parameters for image acquisition were kept constant between samples. Scale bar, 20 μm. (C) DAOY cells were incubated with increasing concentrations of ouabain or gramicidin A and 100 nM TSA. Posttranslational modification-specific antibodies for tubulin were used for immunoblotting as indicated. (D) Control or HDAC6 knockdown cell lines were incubated with increasing concentrations of A23187 for three hours. Immunoblots were performed as indicated.

Supplementary Figure 2. EGF treatment increases intracellular sodium. (A) DAOY cells were loaded with CoroNa Green in the presence of ouabain. Cells were incubated with EGF or vehicle, the fluorescence signals were acquired for 12 minutes and then quantified using the Slidebook software. (N=7). (B) DAOY cells were preincubated with PD98059 (MEK inhibitor) or LY294002 (PI3K inhibitor) and then stimulated with ouabain or gramicidin for three hours. The cells were lysed and subjected to immunoblotting with indicated antibodies.

Supplementary Figure 3. Sodium-induced acetylation of tubulin and calcium signaling. (A) DAOY cells were pretreated with vehicle or 10 μM KN-62 for one hour and then EGF (10

ng/ml) was added for indicated time points. Immunoblots were performed with indicated antibodies. (B and C) DAOY cells were incubated with either vehicle, 10 μM ALLN, 10 μM calpeptin (B) or 10 μM cyclosporin A (C) for one hour and then ouabain or gramicidin A were added for an additional three hours in the presence of inhibitors. Immunoblots for acetylated and total tubulin are shown.

Supplementary Figure 4. Average vesicle speed in nocodazole-treated cells. Analysis of the average speed of EGF-positive vesicles upon disruption of microtubules with nocodazole in Low Na^{+}; *, $P<0.0001$.

Supplementary Figure 1.

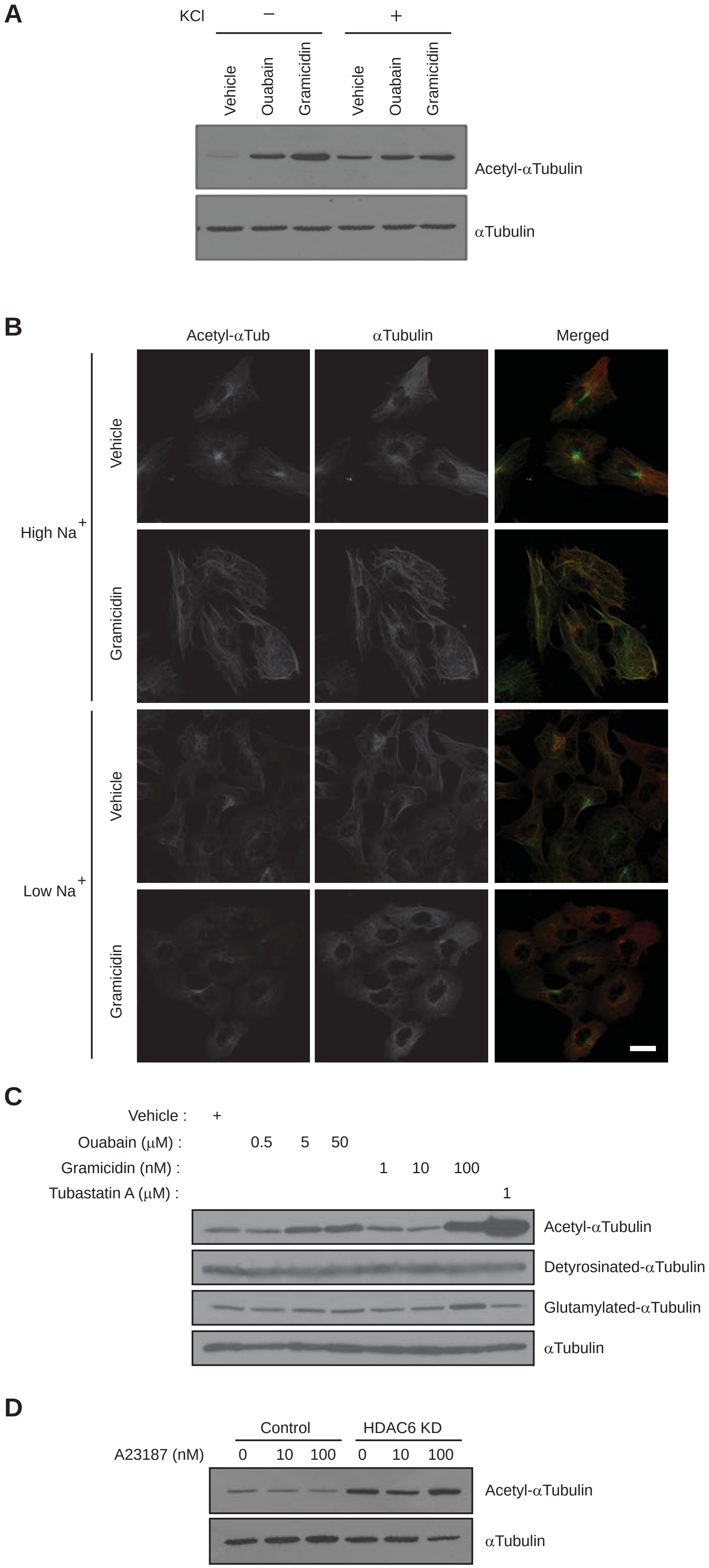

Supplementary Figure 2.

A

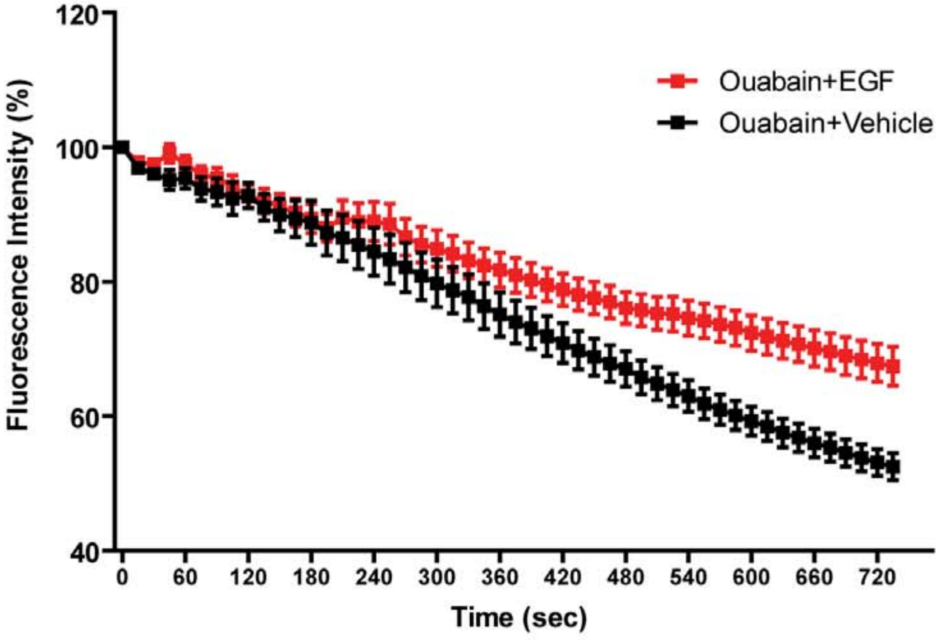

B

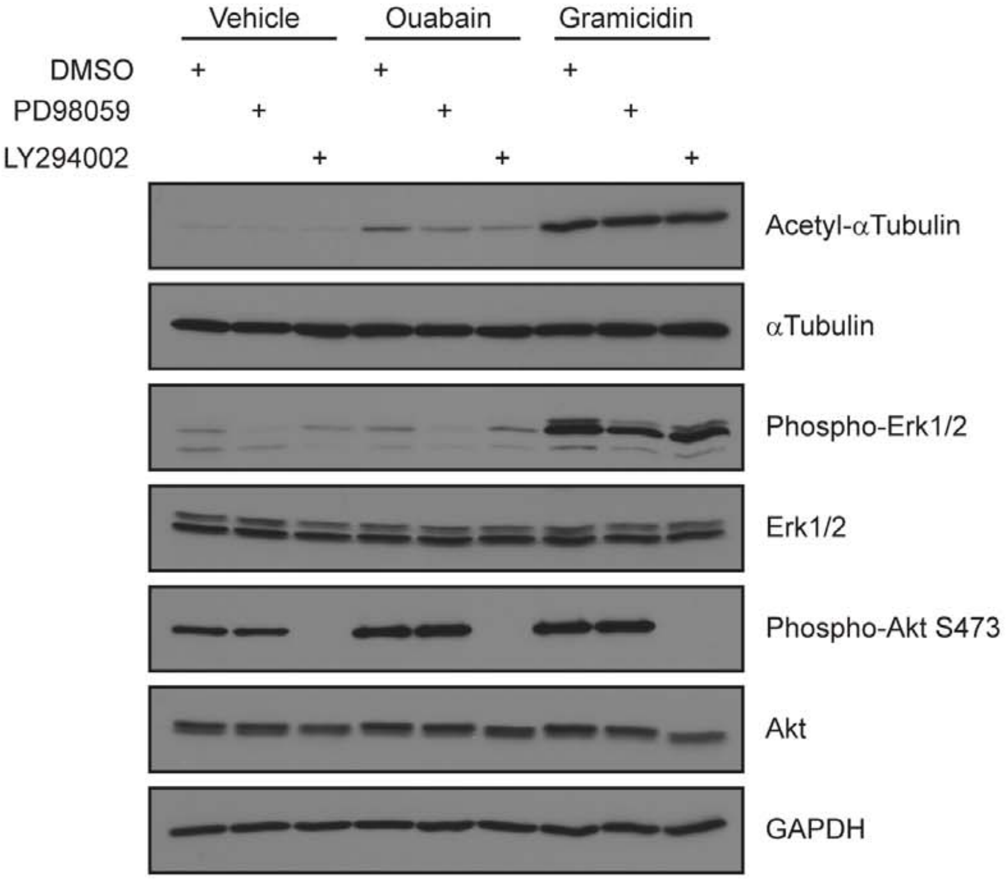

Supplementary Figure 3.

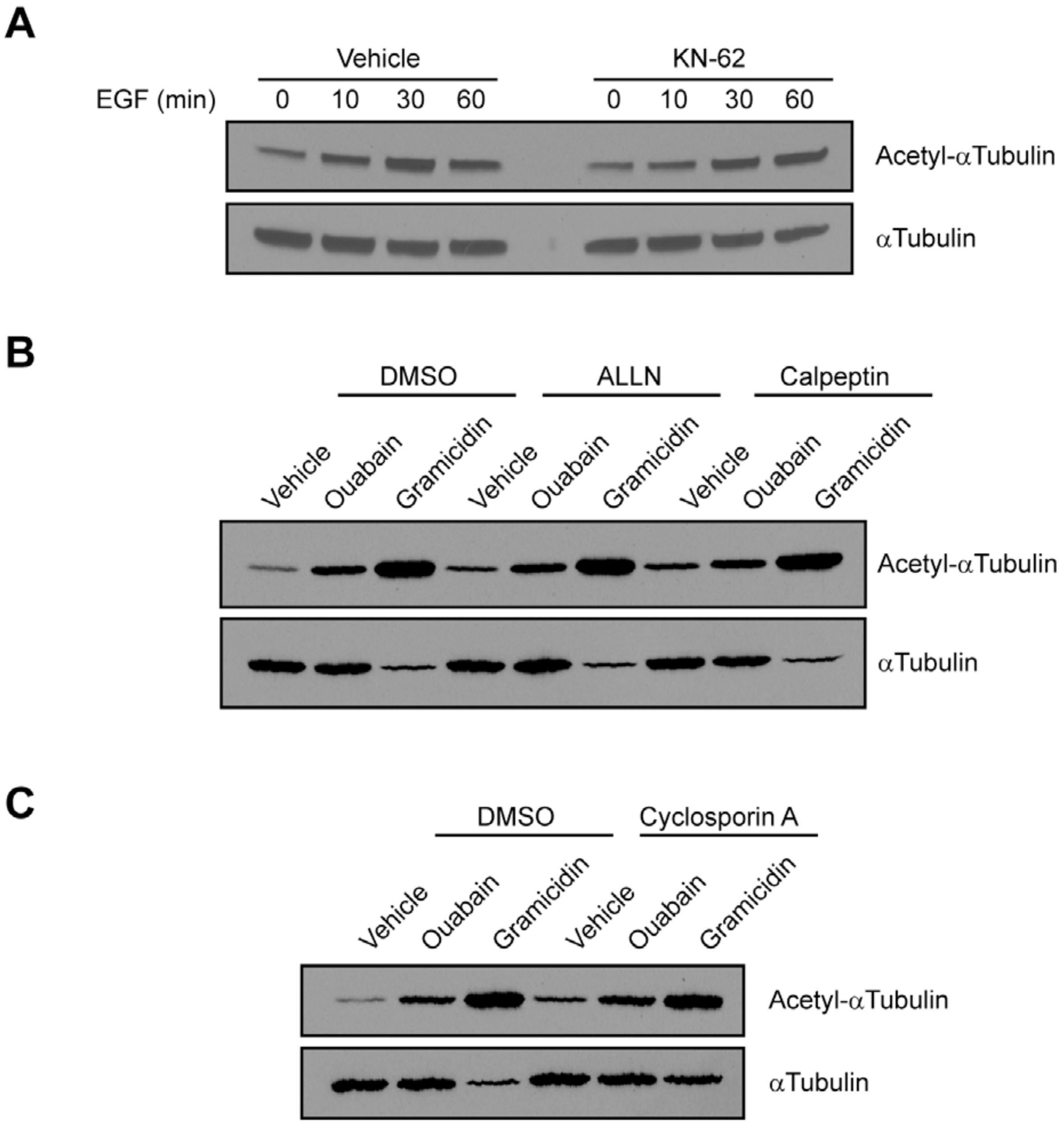

Supplementary Figure 4.

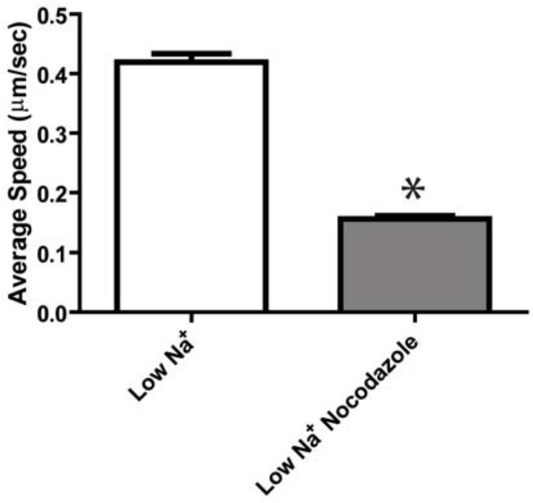

Supplement: Additional file 1: Figure S1. — Sodium-induced post-translational modifications of tubulin is limited to acetylation. Figure S2. EGF treatment increases intracellular sodium. Figure S3. Sodium-induced acetylation of tubulin and calcium signaling. Figure S4. Average vesicle speed in nocodazole-treated cells. (PDF 360 kb) [file 12860_2015_70_MOESM1_ESM.pdf]
